# Should This Tooth Be Extracted?

**Published:** 1917-01

**Authors:** Edgar D. Coolidge


### "SHOULD THIS TOOTH BE EXTRACTED?"

If we accept the statement for the present at least, that there are from seven to twenty per cent. of teeth which have root canals that can not be properly cleaned and filled what are we to do with them and how are we to know when such a statement as "this tooth should be extracted" is the only thing to say? If the future proves that all canals filled to the apex with gutta-percha under strict aseptic conditions are free from infection, areas of bone absorption and other symptoms of local foci then we may conclude that this treatment is all that is necessary and strive to improve our technic enough to narrow down the per cent. of unfillable roots. At this present time this thought is promising and offers the most hopeful solution. However, another problem enters root canal work which has not been considered in the past. When we inspect a number of decalcified teeth the canals of which have been previously stained to make them opaque and the tooth structure made translucent, they reveal many branches of the main canals and often several foramina along the sides of the roots as well as on the end and sometimes have no opening at the very apex of the root at all. A very large per cent. of roots show this condition present, a fact which increases the difficulties attending this work and places more severe requirements upon perfect root canal filling. Unless the organic matter can be removed and the canal thoroughly filled there will always be a danger point at that place. In cases where the roots are bent at such angles that no instruments available at the present time can be successfully used to open the canals it would be better to extract the tooth than leave it to become a future focus of infection. Frequently molars and bicuspids and sometimes any root will show a bad curve in the apical third. If these conditions are located by radiographs before an operation is begun it should be a very strong influence in determining whether or not the pulp should be involved. Many pulps may be saved that in past years have been removed, and every possible treatment for preserving healthy pulps should be encouraged.—Dr. Edgar D. Coolidge, *Dental Review.*

